# Providing social assistance and humanitarian relief: The case for embracing uncertainty

**DOI:** 10.1111/dpr.12613

**Published:** 2022-04-09

**Authors:** Matteo Caravani, Jeremy Lind, Rachel Sabates‐Wheeler, Ian Scoones

**Affiliations:** ^1^ PASTRES Programme Affiliate Rome Italy; ^2^ BASIC Research/PASTRES Programme, Institute of Development Studies University of Sussex UK; ^3^ BASIC Research, Institute of Development Studies University of Sussex UK; ^4^ PASTRES Programme, Institute of Development Studies University of Sussex UK

**Keywords:** conflict, disaster response, humanitarian relief, risk, social assistance, uncertainty

## Abstract

**Motivation:**

Social assistance, humanitarian relief, and disaster response increasingly overlap, especially where recurrent crises and persistent conflicts prevail. In such situations, distinctions between risk and uncertainty become especially important. It is critical for policy and practice to shift from focusing on risk assessment and management to embracing uncertainty.

**Purpose:**

The article assesses the appropriateness of two approaches to social assistance and humanitarian relief where crises recur and conflicts persist: risk assessment and management; and embracing uncertainty and ignorance.

**Methods and approach:**

The article reviews different approaches to social assistance, humanitarian relief, and disaster response, and asks how they are framed. It draws on experiences from programmes offering social assistance, humanitarian relief, and disaster response, highlighting the professional, bureaucratic, and institutional features that influence programme design and functioning. These are compared with “high‐reliability” approaches deployed in other critical infrastructure—such as water and energy supply.

**Findings:**

Mainstream approaches focus on risk assessment and management, assuming predictability and stability. This is problematic, especially in settings of crisis and conflict where there may be no functioning delivery system for social assistance and relief. The article highlights alternatives to the mainstream risk‐focused approaches, which emphasize learning, collaboration, adaptation, and flexibility. Such approaches must build on embedded practices of moral economy, collective action, and mutual care and be supported through professional and institutional capacities that generate reliability.

**Policy implications:**

The article suggests a new agenda for the intersection of social assistance, humanitarian relief, and disaster response, which makes uncertainty the focus for rethinking responses at scale, especially in settings affected by crisis and conflict.

## 1 INTRODUCTION

Delivering assistance to people in crisis‐ and conflict‐affected settings is a major and growing challenge. In such circumstances, practitioners must grapple with contexts at the nexus between the standard delivery mechanisms of state‐led social assistance and the approaches of humanitarian relief (cf. Buchanan‐Smith & Maxwell, 1994; Roelen et al., 2018).

In such contexts, a whole array of uncertainties affects implementation, resulting from fractured authority, conflict, or disaster conditions. On the ground, such uncertainties must be negotiated on a daily basis, and often informal professional and operational practices emerge in order to deliver assistance reliably. These occur among local‐level public authorities (such as elders, religious figures, women, youth leaders etc.), as well as among field‐level implementers involved in co‐delivery arrangements of formal programmes. But these practices, even when they are widely acknowledged to be happening, often exist in tension with prevailing policy and institutional frameworks that assume stability, control, and the ability to manage risk through a functioning formal system led by governments and aid agencies.

This article explores this tension, arguing that it is essential explicitly to recognize uncertainty and acknowledge the practices followed by diverse actors that generate reliability in the delivery of social assistance in crisis‐ and conflict‐affected settings. We argue that embracing uncertainty can help us rethink the intersection of social assistance, humanitarian relief, and disaster response in fundamental ways, resulting in a greater alignment of on‐the‐ground practice with wider policy and institutional approaches.

At the heart of this tension is the important distinction between risk—which is predictable and therefore manageable—and uncertainty, which is not. As the economist Frank Knight (1921) noted long ago, risks are when future outcomes are known or can be predicted, while uncertainty is when we do not know the probabilities of such outcomes. Further, ignorance is when we do not know what we do not know: where neither outcomes, nor their probabilities, are known (Scoones, 2019. These basic distinctions are important, as our knowledge about the future influences how we act in the present. Under conditions of uncertainty, the gap between assumed risk and actual conditions is large, as non‐linear, telecoupled interactions always increase in highly complex, turbulent systems (Liu et al., 2013). Uncertainties and sources of ignorance in turn expand and the ability to predict, manage, and control inevitably diminishes. This is the case in nearly all settings where social assistance and humanitarian relief are delivered under conditions of crisis, disaster, and conflict.

Uncertainty and ignorance are the dominant features in settings where state capacity is weak; where responses by international aid organizations are patchy; where trust and public confidence are low; where infrastructure is inadequate or destroyed; and where insecurity disrupts operations. In such operational settings, the core challenge thus becomes to embrace uncertainty and confront ignorance in order to deliver assistance to those affected by crisis and conflict in a reliable manner. This results in enabling affected populations not only to withstand and “bounce back” from crises, but also to navigate and, where possible, exploit a range of uncertainties, and so avoid the dangers of ignorance and complete surprise.

Assuming that we know about the future, or that it can be predicted through models or other means, results in a risk‐focused approach where implementation responses are centred on control and top‐down management (Scoones & Stirling, 2020). However, when future risks cannot be predicted and events are often surprises, the focus must be on generating reliability—a guaranteed supply of services or ability to provide assistance—in the face of uncertainty or ignorance. Where operational contexts are subject to high levels of unpredictable variability—we would argue the most common ones faced by most practitioners working in conditions of recurrent crisis and protracted conflict—a focus on risk prediction and control is inadequate. Instead, we contend that there is a need to explore a “high reliability” approach that recognizes the importance of uncertainty, and does not close down to a managerial, control‐oriented approach.

Between the bookends of humanitarian relief or disaster response and more regularized and shock‐responsive social assistance, there is a complex reality: an increasingly common messy middle consisting of situations of crisis that are neither stable enough for “development” nor in permanent “emergency.” Indeed, much of the world exists in such shades of grey between these extremes, where conditions are turbulent, crisis is normalized, and public authority is both contested and fragmented, especially in areas experiencing protracted conflicts (cf. Winder Rossi et al., 2017; Sabates‐Wheeler, 2019).

This article therefore argues that uncertainty must inform the conceptualization and operationalization of social assistance in settings affected by crisis, disaster, and conflict, resulting in a rethinking of institutional and policy frameworks and a recognition of the on‐the‐ground practices of practitioners who already by necessity must negotiate uncertainties and generate reliability. This has significant implications for the design of programmes, and so for training, staffing, funding, and styles of accountability.

## 2 RISK ASSESSMENT AND MANAGEMENT: A DOMINANT APPROACH

Why is it that approaches centred on risk assessment and management have tended to dominate mainstream approaches to social protection and humanitarian assistance, even in conflict settings?

Social assistance approaches within wider social protection programmes have largely emerged in relatively stable settings where long‐term government or donor‐funded assistance is provided as a predictable cash, voucher, asset, or food transfer, or in exchange for labour for public works. Such assistance aims to smooth consumption or income and so allow households to survive, and possibly to innovate and invest to boost their livelihood chances. These approaches often require the establishment of managed, regularized information, payment, and targeting systems that assume stable, predictable futures.

The World Bank's social risk management (SRM) framework is an example of such a risk‐based approach (Holzmann & Jorgensen, 1999). Proponents argue that identifying strategies for dealing with defined risks—almost exclusively economic risks associated with income and assets—allow for greater prediction for operational delivery. Updated in 2019 with a wider acknowledgement of uncertain disruption, the SRM approach remains firmly wedded to a risk‐assessment and management framing (Jorgensen & Siegel, 2019). The same is true of other approaches to social protection, such as the life‐cycle approach to programming advocated by the International Labour Organization (ILO) and the United Nations Children's Fund (UNICEF) or the social protection strategy of the World Food Programme (WFP) focused on managing defined risks (WFP, 2021).In all these approaches, risks are attributed either to an individual characteristic (idiosyncratic risk) or to an exogenous factor with wide effects (co‐variate risks), and the focus is on the prediction, management, and control of known risk events through centralized and (state‐led) formal systems.

A risk‐focused approach is central to the operational challenge of “targeting” common to all such approaches, encouraged by demands for efficiency and effectiveness in reaching those in greatest need within operational programming. Targeting is often defined by relying on either quantitative poverty analysis of nationwide socio‐economic datasets or more specific risk and vulnerability assessments. Such approaches aim to predict who is at risk and where, evaluating whether an individual, household, village or even region is currently poor/food‐insecure and/or likely to remain/become poor. Since the early 2000s, poverty‐targeted social assistance programmes have become increasingly widespread, with nearly two thirds of countries in Africa having a social protection policy or strategy by 2019 (Devereux, 2020). Supported by national governments, and usually funded through donor programmes, they have proved successful in many cases, reducing extreme poverty and destitution (Banerjee et al., 2015), including throughout the COVID‐19 pandemic. Yet, as we argue below, they may not be the ideal models for implementing social assistance programmes in areas affected by crisis and conflict.

A focus on risk assessment and management is equally central to much humanitarian assistance. Disaster risk responses are, for example, central to the Sendai framework that governs international efforts in this area. The aim is to develop predictions for expected disasters and in turn plan for the outcomes. It is hoped that more effective modelling and the resulting planning will “dull” disasters and reduce the devastating consequences for those affected (cf. Clarke & Dercon, 2016). Improved prediction capacities—including for disasters linked to droughts, flooding, hurricanes and so on—assist with such efforts, but there are limits to “downscaling” such broad climate predictions to specific risk predictions for particular places at certain times (Jones & Thornton, 2013). Some sources of disaster—such as earthquakes and, in turn, tsunamis—are notoriously difficult to predict. As a result, even if predictions are possible, uncertainties prevail and surprises are inevitable (Hough, 2016).

Where social assistance is being delivered in the context of conflict, similar challenges arise. Invariably, conflict is treated mainly as an exogenous risk that disrupts an otherwise peaceful environment. Attempts are frequently made to predict conflict risks through elaborate modelling approaches (e.g. Delany & Varga, 2002; Cederman & Weidmann, 2017), but problems arise when attempting to predict what is so often completely unpredictable. Spatial mapping of conflict events, such as by the Armed Conflict Location Event Database (ACLED), exposes longitudinal trends; however, given the focus on *post hoc* reporting, the predictive capacity is inevitably insufficient to define interventions. Leaving aside the limits in the availability of data or access to places experiencing acute violence, the very nature of conflict dynamics, involving unforeseen interactions among an array of actors, simply does not allow for the prediction and precise forecasting of particular events, and uncertainties and often ignorance prevail.

As any field‐based practitioner will admit, there are always multiple factors that cause even the best‐designed social assistance or humanitarian programme to unravel. A combination of events may befall a particular family—through a death or illness, for example—such that, through a series of cascading uncertainties, they are suddenly tipped into extreme vulnerability, unpredicted by any generic assessment model. An unexpected conflict, a plague of locusts, a disease outbreak, or any other surprise event may equally impinge, upsetting the carefully calibrated size and modality of response. While the standard methods of targeting and delivery highlight areas of need, they are never precise, and there is always a need for adaptive flexibility and contingent responses in actual implementation. This is especially the case in situations of conflict or sudden‐onset disaster, where uncertainties and sources of ignorance inevitably expand. For this reason, contingency funds become crucial tools in such settings, where predictions and prior plans fail.

As social assistance, humanitarian relief, and disaster response navigate their overlapping remits, approaches such as “shock‐responsive social protection,” “anticipatory action,” and “adaptive social protection” are attempts to find ways to respond to co‐variate shocks affecting everyone in the same area (Davies et al., 2008; O'Brien et al., 2018; Wilkinson et al., 2020). However, most applications of such approaches remain firmly embedded in a risk‐assessment and management framework, even where flexible adaptations occur on the ground through vertical and horizontal adjustment of operations. The focus on “shocks,” for example, highlights events—moments of disruption and crisis—around which mobilization of resources and actions must occur. The emphasis is not just on post‐shock response, as forecasting and anticipation of events is a key part of the approach (Wilkinson et al., 2018) and central to building prior resilience (O'Brien, 2020). Events are predicted as risks, rather than seen in a wider context of unfolding uncertainties, and anticipatory actions therefore can be predefined and prepared for in a proactive response strategy.^1^ Further, and contrary to many situations in practice, it is assumed that a state‐supported system of assessment and response is in place to identify the beneficiaries quickly and scale up coverage or the size of transfers as required.^2^

However, in many fragile and conflict‐affected settings, or where disasters have destroyed physical and organizational infrastructure, responding to uncertainties and sources of ignorance, rather than relying on prediction, anticipation, and risk management, becomes especially important. As we argue below, this requires a rethink of standard approaches, encouraging a more explicit focus on generating reliability under conditions of uncertainty and ignorance as part of the wider discussion of “shock‐responsiveness,” “anticipatory action,” and “disaster risk management” (Aly, 2021). Before suggesting a way forward, we must first ask why it is that most mainstream approaches continue to resist such a leap, and remain firmly embedded in a risk‐assessment and management framing. The next section explores some of the reasons why.

## 3 PROFESSIONAL, BUREAUCRATIC AND INSTITUTIONAL BIASES

For many organizations, the Holy Grail is a well‐planned, predictive system that eliminates uncertainty, ignorance, and surprise in addition to managing risks where probabilities are known. A well‐respected organization focused on disaster responses, for example, argues in its vision statement that it is founded on:

“…the principle that the relative likelihood of particular disasters can be predicted, and that their impact can be managed, with the right plans in place…. because we believe that disasters should not be surprises.”^3^

There are several factors that entrench such a risk framing in mainstream institutional and policy approaches. Prediction, and so planning and management, allow for organizations to manage budgets, personnel, and activities in ways that are feasible—especially for large, hierarchical organizations, such as government departments, large non‐government organizations and United Nations agencies, which dominate the social assistance landscape. Planning and management are seen to be enhanced through a suite of risk‐assessment approaches, based on needs assessments, early‐warning systems, anticipatory modelling, trend analysis, and data‐mining. Even if never accurate, the sense of being in control is important. Globally, large amounts of money are in turn invested on the assumption that such systems can improve the efficacy and efficiency of delivery of social assistance and humanitarian relief.

In cases where prediction is possible and stability and control the norm, and there are sufficient funds and a functioning organizational infrastructure, such approaches have much merit, and form the basis of successful social assistance programmes. Responses to COVID‐19, for example, have provided social protection for a great many people through new programmes with significant funding. However, many of these have been stop‐gap measures that have not recast the way such programmes respond to uncertainties (Leisering, 2021). Especially when external funds dry up, there is a significant mismatch between risk‐based prediction—and the resulting plans and programmes—and the outcomes on the ground.

Rather than questioning the assumptions behind risk‐based planning under conditions of uncertainty, however, the mismatch is often put down to “implementation failure,” “poor governance,” “lack of co‐ordination,” “lack of funds,” “political economy effects,” or a range of other design and implementation faults. These failings are, of course, more often identified in poorer settings where uncertainties are rife and where implementation systems are weak. Rather than focusing on the “failure of implementation,” within parameters that are impossible to achieve, it is perhaps more honest to question the premises of the intervention, and ask whether there are other ways to achieve reliable delivery in the given context.

There are a number of professional, bureaucratic, and institutional biases that reinforce such a risk‐management and control approach, rather than encouraging an opening up to uncertainty. As already discussed, in relation to social assistance programmes, there is much emphasis on targeting and the efficiency of the delivery system. Many programmes have been funded and designed by aid agencies, whose upward accountabilities require intensive forms of audit. Fears of corruption, elite capture, and patronage—common among many government social welfare programmes the world over—mean tight assurance and accountability protocols and intensive regulation against “leakage” and “inclusion errors,” whereby households and/or individuals are included even though they do not meet poverty‐defined targeting criteria. A restrictive “audit culture” (Power, 2004) prevails, and flexibility and adaptation to particular circumstances are frowned upon, as those in charge of the monitoring and evaluation systems (including grievances and redress mechanisms) are not allowed to account for deviations.

Targeting inevitably makes a number of assumptions. Local understandings of “need” and being “at risk” may differ quite radically from those used by external programme designers, based on particular metrics, such as poverty lines, asset ownership, or food production “gaps” (Pruce, 2019). Interventions usually target an individual or household and do not appreciate the wider collective dynamic of mutual assistance and support. Co‐variate shocks are continuously addressed by people and groups within communities; not just at an individual or household level, but among families, kin groups or villages. Here forms of sharing and redistribution—both formal and informal—become important, as part of a wider “moral economy” (cf. Scott, 1977), which is not encompassed in the individual/household delivery systems of standard programmes focused on risk‐based, needs‐based targeting.

Humanitarian assistance in disaster responses again aims for more accurate anticipation through prediction and preparedness in order to prompt early responses. Much effort is spent on contingency planning based on alternative risk scenarios, derived from different models. As Clarke and Dercon (2016) argue, effective disaster planning responses must “think like an insurance company,” with actuarial logics and linear planning to the fore. Organizations involved in disaster response rely on expertise in planning and logistics, where protocols are strict and flexibility reduced to the minimum to ensure streamlined operations. The focus is on a prospective, single event—a disaster that can be declared as a “crisis” or “emergency.” For humanitarian organizations requiring funding to be released this is crucial, and delays in declaring a crisis or emergency cause many problems with logistics and delivery to often challenging settings (Levine et al., 2020).

Across social assistance programmes there are, therefore, various professional, bureaucratic, and institutional biases that reinforce a control and management‐oriented risk response. The ideal is seen as a stable state and return to conditions of predictability, helped through the implementation of sequenced interventions and expert inputs. The focus on predictive modelling of risk and need emphasizes a quantitative approach, where accredited knowledge processed by experts and derived from surveys, models, and formal scenarios is seen as more authoritative than qualitative, locally generated, informal, and tacit, experiential knowledge of field‐based staff or crowd‐sourced analysis generated by crisis‐affected populations themselves. The appeal of high‐tech satellite‐image‐based predictions, data‐mining or complex actuarial models is that they give a sense of control, even if uncertainties are everywhere. Planners, logistics experts, and project managers are trained to work to clear protocols, with quantitative indicators of delivery and achievement, and again erring to accommodate uncertain change is seen as unprofessional, even illegal. The bureaucratic requirements for upward accountability have been reinforced by “new public management” reforms across organizations. The watchwords are efficiency, lean delivery, and a results orientation, governed by neat log‐frames and planning protocols. Audits of every aspect, from procurement to delivery to distribution, are standard and the discretion and room for manoeuvre of those involved in these processes is minimized through regulations (Eyben et al., 2015).

The result is that implementing organizations frequently lack flexibility and the ability to experiment, improvise and fail (Ramalingam et al., 2008). Again, the focus is on “projects” and “events,” centred around particular crises and with expectations to demonstrate performance and quantifiable results, frames the challenge in terms of delivery in linear time, rather than responding more openly to unfolding, uncertain temporalities, as experienced by people on the ground (Anderson, 2010; Bennett, 2018). Within bureaucracies, the competition over funds, prestige, and control also undermines flexibility. Funding agencies like a quick‐fix, technical solution, especially one dressed up in the language of “innovation,” “value for money,” and “return to investment,” but they often will only pay for things that appear to offer certainty, where outputs and outcomes can be measured in relation to specific metrics.

All these features are affected by wider, cultural, institutional, and political factors. The imperative to respond to the assumed “needy” poor in an efficiently targeted way emerges from a particular style of individualized, market‐driven implementation. The mantras of efficiency and audit emerge from a sequence of economic and management reforms over the past decades that have generated certain types of institutional response in aid agencies and governments alike. Cash‐starved governments and international humanitarian and aid agencies rely on crises or emergencies to raise funds or justify their expenditure. This in turn undermines funding commitments in the periods between crises or between electoral cycles when the investment in preparedness capacity and building the routines for addressing uncertainties and uncovering sources of ignorance must happen.

Of course, despite these biases in favour of a controlled risk‐assessment and management approach, in practice, the reality on the ground is often different. Such strictures are never as extreme as the formal institutional design or policy edicts suggest in conditions where field‐based practitioners must navigate contexts in real time. But, nevertheless, flexible room for manoeuvre is constrained by prevailing frameworks and assumptions, which ignore the more complex realities faced by social assistance and humanitarian practitioners, especially in disaster‐ and conflict‐affected settings. The mismatch between the assumed design and actual practice is often stark.

What, then, would a radically different approach look like that with uncertainty, ignorance, and surprise, rather than calculable, manageable risk, as the starting point? The next section explores some key principles through a brief reflection on “high reliability management” approaches.

## 4 EMBRACING UNCERTAINTY: HIGH‐RELIABILITY APPROACHES

Drawing on the field of “high reliability management” as applied to “critical infrastructures” (Roe & Schulman, 2008; Roe, 2016), we ask how we can embrace uncertainty and confront ignorance in the implementation of social and humanitarian assistance programmes—delivering services reliably—especially in crisis‐ and conflict‐affected settings.

For critical infrastructure—such as water and energy supply systems—as with social protection and humanitarian assistance, the standard approach is to calculate risks, plan on the assumption of predicted futures, and exert more control through stable management regimes—more protocols for safety, stricter regulations, improved standards, enhanced technology, efficient management systems, and better co‐ordination. But, based on a detailed understanding of what actually happens and how services are delivered and safety is assured, the assumptions of a risk‐management approach are challenged. In practice, reliability is generated from highly variable input conditions, where uncertainty is everywhere and ignorance a real threat through managing “mess” and complexity. This occurs in real time by networks of professionals (“control room operators” and their allies) who are able to assess the wider system dynamics, while addressing day‐to‐day operations, and so adapting flexibly to manage uncertainty and avoid ignorance (Roe, 2016). While of course such critical infrastructure settings are not exact equivalents of social assistance and humanitarian response systems, the objectives of assuring reliable delivery (in this case of social assistance) under highly variable, uncertain conditions remain. Four themes emerge from the high‐reliability management literature that, we argue, have wider relevance.

The first is the role of what are termed “high‐reliability professionals.” Such professionals must move between the “nuts and bolts” of the system and wider horizon‐scanning and modelling of unknown futures. In the process, they make sense of complexity, experimenting, adapting, and responding quickly. And, in so doing, they must respond to uncertainties, while avoiding the dangers of ignorance and complete surprise. They are not just single individuals operating alone, but exist in networks, within and outside a core implementing organization, working together to co‐deliver safe and reliable services. They do not follow a fixed plan, but have to communicate, learn, and improvise. They frequently go unacknowledged, as within organizations they may be low down in a hierarchy and have a job description that does not match the importance of what they do; and, outside the core implementing organization, they are often hidden, yet, together with others, they are crucial for keeping the system safe and reliable, despite massive variability in input conditions.

Second is the importance of accumulated learning and sharing of information across and between peers within networks. High‐reliability professionals are key to this, and crucial in generating knowledge within organizations. No single plan or protocol is sufficient, as what happens in one place will differ from another. This means that organizations must embrace experimentation, learning, and improvisation. Learning from (non‐catastrophic) failure can help in this process as networks of professionals navigate uncertainty together, accumulating experience along the way. Such experiences may result in innovative practices and procedures, developed from the bottom‐up in ways that become vital for assuring safety and reliability. Such practices should not be seen as a diversion from a fixed plan or protocol, but rather as integral to enabling more effective responses, and so should be celebrated, not punished or hidden from view.

Third is the observation that reliable systems that can respond to uncertainty are neither formal nor informal, but usually hybrid combinations. While formal systems provide a broad infrastructure, it is locally embedded networks within organizations at the operational coalface that become important for processes of learning and adaptive response. Such networks are also crucial for building trust and capacity within organizations, making it possible to respond rapidly to unfolding circumstances if a potential disaster emerges. Such local networks may involve multiple people with personal connections linked across formally defined organizational divisions. In such settings, accountabilities are downwards to local systems and horizontal across networks that are brought together and are not located in a single delivery organization. Thus, in high‐reliability systems, networks are central, as people must link across domains and between organizational mandates.

Fourth, organizational and funding flexibility is essential to reliable responses in the face of uncertainties. Since futures remain unknown, fixed budgets, standardized plans, and uniform protocols are inappropriate. Instead, plans provide frameworks not diktats and contingency plans, and anticipatory financing can allow rapid response, as long as these remain flexible and not linked to pre‐defined risks. Some level of system redundancy within an organization is important too, with slack taken up when challenges arise, as new people, funds, and capacities are deployed. Funding arrangements that are not tied to fixed outputs and elaborate systems of control are essential, allowing for contingent, flexible, and rapid responses by people within networks who know what is happening and can do something about it in real time.

How do these themes, widely discussed in literatures on critical infrastructures and high‐reliability management, apply to examples where support through social assistance and humanitarian relief combine in contexts that are highly uncertain and affected by conflict? In the next section, we explore this question through two cases.

## 5 CONFRONTING UNCERTAINTY IN THE FIELD

In this section, we examine two cases in which uncertainties are confronted in the implementation of social assistance and humanitarian relief programmes where protracted conflict and high levels of uncertainty are prevalent. The first is the Ethiopian social assistance programme, the Productive Safety Net Programme (PSNP), originally designed for the agricultural highlands and now being implemented in the conflict‐prone (agro‐) pastoral lowlands. The second case is the *Zakat* Fund in Libya, being implemented in a context of continued conflict, with fragmented and contested state authority. Both programmes illustrate how the themes highlighted from the high‐reliability management literature are central; at least in the day‐to‐day practice of such programmes if not always recognized by formal plans and designs.

### 5.1 Adapting a safety‐net programme in Ethiopia's lowlands

The lowlands of eastern Ethiopia, in Afar and Somali administrative regions, present a challenging context for the delivery of services, including social assistance. These predominantly pastoral rangelands have a fraught relationship with national government, with warfare, insurgency, and more everyday subversive resistance characterizing state–society relations in the past. Ministry planners have long struggled to adapt models of service provision to the social and governance contexts in these areas, where the degree of “statehood” blends with non‐state, customary public authorities.

Since 2010, Ethiopia's national government has expanded the reach of the PSNP into the lowlands. The PSNP is one of Africa's largest social‐transfer programmes, providing monthly payments of cash and food to more than 8 million households. To a large extent, planners sought to transfer the PSNP delivery model unchanged to the lowlands: district (*woreda*), sub‐district (*kebele*) and community‐level Food Security Task Forces (FSTFs) were established to oversee targeting of households and provision of regular food transfers; *woreda*‐level committees were assigned responsibility to implement public works projects that drew on the labour of PSNP beneficiaries, and *kebele*‐level appeals committees (KACs) were set up to consider grievances and complaints.

Evaluations of the PSNP in the lowlands have consistently shown that the programme has underperformed compared to highland agricultural regions. Perplexed by this, official responses initially focused on extra efforts to strengthen training for lower‐level implementers and monitor more closely the targeting of the programme. Quantitative data from evaluations has shown that the poorest are often some of the least likely to be included, with those who are better‐off consistently being covered instead. Formally mandated targeting procedures were being reinterpreted on the ground such that transfers were being distributed much more widely than had been officially planned for. This knowledge led to a revision of targeting guidelines explicitly to incorporate the involvement of local traditional leaders—assumed to be the most knowledgeable about who is in need and hence to correct targeting errors. Despite this, there was no observed change to the pattern of the better‐off being included.

Despite the central planners' assumptions of capacity shortfalls, lack of understanding and/or outright favouritism by local traditional leaders in these areas, closer inspection of decision‐making by local‐level implementers point to more complex dynamics at work. Part of the implementation challenge in the lowlands is that levels of need greatly outstrip the programme's intended coverage. Hence, local officials face extremely difficult decisions about who to include, especially when the difference between the extreme poor and the poor is so marginal:

“…judged by vernacular understandings of need and culturally embedded notions of mutual obligation, it is not an ‘error’ to include households who are marginally better‐off — at least according to asset‐based measurements—but rather a justifiable intent to extend coverage” (Lind et al., 2022).

From the perspective of managing uncertainties, the “reliability professionals” are therefore local leaders who make use of vernacular understandings of risk and need, conditioned by understandings of fairness, in order to fill the gaps in provision. Furthermore, they do not act entirely separately from frontline, lower‐level programme implementers. Sometimes they sit within FSTF and KAC structures; sometimes they are positioned adjacent to these, helping to innovate around an adapted form of delivery and, with good knowledge of the context, were able to be inclusive, even if they themselves were largely older, male elites in the community.

If the central planners who designed the programme for a highland setting appreciated these dynamics, then the outcomes of redistributing transfers can be seen very differently. The way that protection of the lives and livelihoods of the poorest plays out on the ground cannot be dictated by universal norms and blueprints set by policy‐makers and donors. Rather, access to social provision is negotiated in context and is imprinted by local values of care and responsibility. Many high‐reliability responses are therefore centred on shared, collective understanding and action, even if these arise from starkly different positionalities and values; what in organizational studies is called “situational awareness,” but in other settings might simply be referred to as community‐based local knowledge and moral economy, going beyond the individual or household.

Of late, lessons from lowland settings to inform the expansion and adaptation of the PSNP has risen to the policy‐makers' attention. Ideas for locally informed and creative use of flexible funding, such as the *woreda* contingency funds, to address unforeseen shocks should have been raised. Equally, innovations for adapting targeting in the lowlands to follow a community approach that accounts for cultures of sharing or the demographic implications of practices of polygamy, for instance, are likely to be better suited to the context, and so more likely to be able to generate reliability in the face of intersecting uncertainties.

### 5.2 Transforming a top‐down welfare system to a networked arrangement: The case of the Zakat fund in Libya

Decades of a socialist regime (*Jamahiriya*) in Libya resulted in national public assistance programmes providing universal food and fuel subsidies, as well as free health care, child benefit, and education. Prior to the conflict in 2011, the social protection system cost 4.4% of the gross domestic product (GDP), financed mostly through oil revenues (IPC‐IG, 2018). However, after 2011, owing to conflict, political instability, and resource constraints, the state‐funded social protection system has largely collapsed (Caravani & Kryeziu, forthcoming).

Alternative forms of assistance have become necessary. *Zakat*, for example, is a widely used form of Islamic charity (Machado et al., 2018), and this has become institutionalized, linking community members and religious leaders with the state. In 2012, the *Zakat* Fund (*sunduq al‐Zakat)* was officially established as an independent entity, and so operates outside the jurisdiction of the Ministry of Social Affairs and autonomously from the two governments operating within Libya (Cabinet of Libya, 2012). Across the country, there are 52 offices that collect *Zakat* from the wealthy, and in turn register, enrol, and target payments, with transfers being both in‐kind and cash.^4^ In 2020, 42,631 beneficiaries received around USD 12.4 million (Caravani & Kryeziu, forthcoming).

In order to function under conditions of fragmented and contested authority resulting from sustained conflict, the *Zakat* system operates through religious networks under Sharia principles. Formally, the *Zakat* Fund leadership is connected to both government parties so that they can work all over Libya, including having access to conflict areas for food relief convoys during Ramadan. For instance, in May 2020, during ongoing conflict in Tripoli, the *Zakat* Fund financed two truck‐loads of basic food commodities for the city of Sabha in southern Libya, which is administratively under Tripoli, but militarily and financially supported by the Eastern government as part of a campaign called: “Be indulgent and you will receive mercy”.

At the local level, mosque leaders and Imams use their networks to reach the local population. They identify the neediest in a particular area and then connect them to the local *Zakat* Fund office. At national and local levels, such leaders act as brokers and facilitators, creating networks and so generating reliability in assistance delivery, often under extremely challenging and uncertain conditions. Such networking in assistance delivery is in turn facilitated by technology. For example, telephone hotlines have been established across the country, launching a programme called “show me the poor” (*dolani ala alfakeer*), whereby on a voluntary basis citizens call and inform the office about the poorest people living in their neighbourhood or village. This programme identified the neediest, who often remain invisible to any forms of social assistance.

Learning about populations helps refine the assistance programme, which is further facilitated by a network of young volunteers, street‐based “social researchers” (*baheton ajtemaeyon*), who are known for their integrity and honesty. They are all connected to the local mosque and Imam and visit the people that have been identified, confirming their eligibility for *Zakat* assistance. Social researchers use their own means to move across the designated areas with a questionnaire to be filled by each person. This is then delivered to a committee based at the local mosque, which decides whether the person is eligible for assistance and on the type of assistance, whether basic food, housing rent, medicines and health care, clothes and blankets, and school materials. Each social researcher is responsible for four areas where they know most of the families living there and stay in phone contact. According to one interviewee (Tripoli, October 2021), at times, social researchers act independently because they are now recognized as local leaders who may receive donations directly.

This social intelligence and learning combined with networking building and brokering by the reliability professionals at the centre of the operation allows for adaptive response in the face of rapid change and uncertainty. Navigating conflict and local politics and operating outside either of Libya's government structures also helps in gaining legitimacy and so ability to function. For example, in April 2021, in the area of Ras Hassan in Tripoli—which, due to the conflict in the country became populated by many internally displaced persons—social researchers collected basic food items (including meat and vegetables) to support about 35 families. One informant we interviewed in Tripoli in October 2021, told us that while men delivered food baskets door‐to‐door, women targeted those most in need, while also fundraising directly to pay for house rents and medicines.

By contrast to the former, well‐funded and top‐down state implementation of social assistance, the current system is much more variegated and flexible, with downward and horizontal accountabilities to the people it is serving. With much less state funding (currently down to less than 1% of a much‐declined GDP), a hybrid organizational framework has emerged, combining the formal and informal, the mandated and voluntary. Responsibilities are shared between the state and other local institutions, notably the autonomous *Zakat* Fund and local mosques. This spreads the implementation challenge in an extremely difficult setting, linking downwards to neighbourhoods and the congregations of mosques, via local participation in identifying need, and the network of social researchers, all of whom are intimately connected to local areas through clan and family networks.

What has emerged is very far from the comprehensive social welfare bureaucracy that existed before. While facilitated through the state, systems of social assistance must instead rely more on informal relationships and knowledge of communities, drawing on traditional religious obligations as well as collective moral economies and practices. Held together by key reliability professionals—both nationally in the *Zakat* Fund and locally through mosque and community leaders, Imams and the network of social researchers—a flexible and reliable system has emerged that is has proven effective in the face of uncertainty and surprise, and is well adapted to the fast‐changing political situation on the ground.

### 5.3 Reimagining social and humanitarian assistance in uncertain contexts

These cases, like many others, show that in practice the control‐oriented risk‐assessment and management framing of policy is adapted on the ground. But such policies and institutional frames still constrain the ability to embrace genuine uncertainty, acknowledge ignorance, and foster reliability in complex, non‐linear systems. As noted earlier, mismatches between policy and practice cannot be blamed on implementation gaps, targeting errors, exclusion by design, lack of co‐ordination, poor governance, and sometimes vague, black‐boxed notions of “political economy.”

What, then, are the key features of an alternative approach, which draws on some of the themes of high‐reliability management and adapts them to the practices of implementation in social and humanitarian assistance, as reflected in the case studies?

**Skills, practices and capabilities**—If uncertainties cannot be planned away, then systems require the capacity to spot uncertainties, address surprises, and avoid areas of ignorance. As highlighted by the high‐reliability management literature and demonstrated through the cases—whether of local‐level implementers and community leaders in lowland Ethiopia or social researchers and mosque‐based networks in Libya—this requires horizon‐scanning, thinking about the longer‐term future, as well as being aware in real time of unfolding situations on the ground. Crucially, it means tracking between these sources of knowledge and translating the insights into timely action. In both of the cases, high‐reliability professionals—or rather networks of professionals—have the skills and capabilities to do this. Such professionals are brokers and translators, linking and negotiating multiple sources of knowledge. Reflexivity and awareness of both knowledge and the consequences of action are key competences, as is the ability to make use of diverse knowledges, triangulating between them, and being trusted within local contexts (cf. Tasker & Scoones, 2022).

Such skills, practices, and capabilities are central to generating reliability in any complex system, and are as relevant to an unfolding social assistance programme as they are to the humanitarian response to a sudden‐onset disaster event. Building this skill base among staff through training and recognizing these practices in existing operations is an important first step. Alternative ways of working “on the edge of chaos” are needed (Ramalingam, 2013), including a focus on learning lessons from failure as well as success; a reliance on collective understandings and actions; leadership that allows for flexibility and responsiveness; and commitments to trust building and accountability reflected in new organizational forms (Ramalingam et al., 2020). In different ways, both the cases offered elements of these characteristics, yet much remained improvised and not always central to official plans and formal designs.

**Organisational change**—Organizations that foster reliability must embed this sort of reflexive, systemic learning that reliability professionals and networks offer. This must be incentivized, supported, and rewarded. When failure offers opportunities for learning, monitoring, evaluation, and reward systems need to recognize this. In terms of organizational structures, a more modular arrangement, with decentralized, networked decision‐making, allows for more nimble, responsive action. The necessary move from a centralized, top‐down system of delivery to a more flexible approach, located in communities, linked to mosques, yet supported through (competing) government structures, was clearly evident in the *Zakat* Fund in the Libyan case. Building in redundancy means that organizations can change gear quickly and have the flexibility to respond if the challenge is larger than expected. Innovation is central to responses to emergencies (Obrecht & Walker, 2016), as one size does not fit all circumstances. Allowing for innovation—and learning from and sharing this—is essential, as is beginning to happen through reflections on the experience of the PSNP in lowland Ethiopia.

Reliability professionals, operating between top‐level senior management and field‐level agents, are seen as vital. The cumbersome, hierarchical organization of a standard government or development or humanitarian agency therefore has to be rethought so that responses are effective and timely. Willingness to adapt and change when things go wrong is important, and this in turn requires effective “adaptive leadership,” which fosters trust through openness, candour, and transparency (Ramalingam et al., 2014). Ideas of “network humanitarianism” for example encapsulate some of this thinking, including an emphasis on new institutions, relations, and behaviours made possible by new technology platforms, incorporating “modular” ways of operating, with in‐built redundancies, and smaller, more specialized units being able to act more nimbly and collaboratively in response to crises (Currion, 2018).

**Financing mechanisms**—Standard approaches to funding are notoriously poor at addressing uncertainties. Single, predictable funding pipelines linked to fixed budgets, planned according to defined risks and negotiated on assumptions of stability and uniformity, are inadequate for unpredictable shocks and surprises. So too is the disproportionate command of humanitarian funding channels by a few mammoth organizations whose control of resources ensures their continued political dominance (Currion, 2018). Financing innovations in social and humanitarian assistance in disaster‐ and conflict‐affected settings include contingency funds, advance commitment funding, and contingency credit arrangements, linked to emergency funding reallocation systems (Centre for Disaster Protection, 2021; Cichon et al., 2004; Clarke et al., 2015; Clarke & Dercon, 2016). The aim is to provide incentives to invest in preparedness on the expectation that surprises will occur, rather than waiting for a disaster to happen, and then relying on (cheaper, maybe free) relief aid, which is often late and inadequate. Investing in such systems is expensive and, as discussed above, creates redundancy in the system, but in the longer term is considerably more effective.

While large‐scale institutional grants will continue to be a necessary mechanism for financing responses to the largest crises, the leveraging of networked technologies opens up other lower‐cost and agile strategies to address the gap in financing responses for other emergency settings (Currion, 2018). Adaptations to standard programmes also include the creation of national and local contingency funds to allow social assistance programmes to shift their focus to address urgent new needs, as the PSNP case from Ethiopia demonstrated. This may involve flexibly responding to unexpected shocks through rapid expansion of top‐up payments to existing beneficiaries, as well increasing coverage, “tweaking” programme design and “piggy‐backing” or aligning with other programmes, as advocated by “shock‐responsive” approaches to social protection (O'Brien et al., 2018).

Under conditions of uncertainty in crisis‐ and conflict‐affected settings, this must go beyond anticipatory risk planning and pre‐shock identification of the most vulnerable within a social registry, and must not assume the existence of effectively functioning delivery systems and infrastructure. As was seen for the *Zakat* Fund in Libya, a decentralized approach, rooted in local capacities and organizations, while supported by government structures, offered a route to delivering assistance, even under conditions of conflict. Through the Islamic traditions of *Zakat* this drew on and adapted more collective forms of solidarity‐based support and moral economy. Reflecting the origins of social insurance and welfare systems more generally, such hybrid approaches potentially offer a way to embrace uncertainties together rather than just alone (Johnson, 2020).

**Accountability relations**—Too often in large organizations involved in delivering social assistance and disaster relief, accountability relationships run upwards—from the “beneficiary” to the provider and funder (Cornwall, 2000). Monitoring and evaluation and audit control follows, focusing on the mandated requirements of a single organization. But, if accountabilities are extended downwards and outwards, then other participants become involved. Those delivering and receiving social assistance are as a result no longer just passive recipients, but are actively involved in the response, as in the “social researchers” operating within particular neighbourhood areas or the local leaders and elders in lowland Ethiopia, working with their own clans and communities.

As both cases showed, locally embedded networks, connecting implementing agency professionals with “beneficiaries” can improve reliability in implementation, as well as holding those in charge to account. Supporting livelihoods requires cross‐sectoral exchanges—between agriculture, nutrition, health, housing, infrastructure and more. As the Libya case highlighted, grounded knowledge of local needs by “social researchers” was able to direct interventions appropriately across everything from food to medicines to housing. Operating outside vertical sectoral structures, the mosque committee linked to the *Zakat* Fund could therefore be more agile in responding to unfolding, uncertain circumstances. Of course, as with centralized, top‐down approaches, exclusions may emerge, as selectivity, favouritism, and patronage may occur in a more open, flexible approach; although in some cases such exclusionary processes may be countered by direct accountability relations in a more localized, embedded system.

After‐the‐event evaluations frequently point to “lack of co‐ordination” as a problem, but if networked arrangements and horizontal accountabilities are built in from the start, a more collectively owned approach for combined knowledge and action ideally can emerge. “Success” can thus be defined more broadly around system reliability, evaluated from different standpoints including those facing uncertainties directly themselves. For example, rather than seeing a “gap” in delivery or a set of targets missed, if, as in the cases discussed above, field‐based practitioners and local people find a way of addressing a particular crisis through novel practices and innovations, then these should be acknowledged, rewarded, and shared.

## 6 CONCLUSION

A focus on uncertainty shines a particular light on incomplete (or absent) knowledge about the future, where standard techniques of prediction, anticipation, and preparedness are inappropriate. While acknowledging of course that we know some things in some places for some people, more generally an acceptance that uncertainty and ignorance are central fundamentally undermines our capacity to plan, manage (even adaptively), and so ensure stable, predictable, and targeted outcomes. This has profound implications for the mainstream, risk‐focused approaches to social assistance and humanitarian relief, especially in settings affected by crisis, conflict and disaster.

Embracing uncertainty and accepting ignorance is clearly challenging for standard approaches to social assistance, humanitarian relief, and disaster response, located as they are in a particular set of risk‐focused professional, bureaucratic, and institutional discourses and practices. Reversing patterns of accountability, incorporating new knowledges and actors, thinking about networks and encouraging systematic and reflexive learning all challenge such standard approaches. Ignoring uncertainty—and not accepting inevitable ignorance and surprise—can be misleading, even downright dangerous. Standard risk‐management approaches paper over cracks that can open up, causing major problems. Fixing these by arguing that all that is needed is better planning, implementation, correcting errors, and improving co‐ordination does not deal with the core issue. Instead, as we have argued, a very different approach is needed.

A “high‐reliability” approach, which embraces and works with uncertainty and complexity, has many different features. It requires fundamental changes in everything from programme design, professional practice and skill‐sets, organizational culture and incentives to funding, monitoring, and evaluation regimes. The good news is that such approaches are already central to other systems designed to generate reliability around “critical infrastructures,” allowing for possibilities of cross‐learning and exchange in reforming the architecture underpinning responses at the nexus of social assistance, humanitarian relief, and disaster response. More encouragingly still, the actual practices of those already involved in many existing social assistance implementation efforts—usually hidden, informal, and outside the regularized, accepted practice of standard programmes, involving actors both inside formal agencies and within local communities—can show the way to doing things differently, as the cases from Ethiopia and Libya highlighted.

As global development and humanitarian challenges increase and converge—whether due to climate change, pandemics, conflict, or long‐term chronic deprivation and insecurity—it is time to embrace uncertainty, confront ignorance, and generate reliability through such approaches to professional practices, organizational arrangements, financing, and accountability relations. If not, the failures of mainstream risk‐ and control‐focused interventions across social assistance, humanitarian relief, and disaster response will persist, and uncertainties in crisis and conflict settings will remain ignored and unaddressed—often with disastrous consequences.

## AUTHOR CONTRIBUTION STATEMENT

All authors contributed equally to the article, which is reflected in the alphabetical ordering.

## Data Availability

Data sharing not applicable to this article as no datasets were generated or analysed during the current study.

## Footnotes

^1^
For example, https://startnetwork.org/resource/risk‐brief‐september‐2021

^2^
While focusing especially on climate shocks, adaptive, shock‐responsive systems in conflict‐affected settings may result in aligning new interventions alongside non‐functioning state systems, as in the case of northern Mali after 2012 (cf. O'Brien et al., 2018, Box 15).

^3^

https://www.disasterprotection.org/about

^4^

*Zakat* Social Media Website in Arabic: www.facebook.com/Zakatlibya/photos/pb.393963277365445.‐2207520000../2880461522048929/?type=3&theater.
